# Application of fluoride disturbs plaque microecology and promotes remineralization of enamel initial caries

**DOI:** 10.1080/20002297.2022.2105022

**Published:** 2022-07-27

**Authors:** Qianxia Zhang, Lingxia Guan, Jing Guo, Aiyun Chuan, Juan Tong, Jinghao Ban, Tian Tian, Wenkai Jiang, Shengchao Wang

**Affiliations:** a Department of Operative Dentistry & Endodontics, State Key Laboratory of Military Stomatology, National Clinical Research Center for Oral Diseases, Shaanxi Key Laboratory of Stomatology, School of Stomatology, the Fourth Military Medical University, Xi’an, PR China; b Department of Preventive Dentistry, State Key Laboratory of Military Stomatology, National Clinical Research Center for Oral Diseases, Shaanxi Clinical Research Center for Oral Diseases, School of Stomatology, The Fourth Military Medical University, Xi’an, PR China; c Department of VIP Dental Care, State Key Laboratory of Military Stomatology, National Clinical Research Center for Oral Diseases, Shaanxi Engineering Research Center for Dental Materials and Advanced Manufacture, School of Stomatology, The Fourth Military Medical University, Xi’an, PR China

**Keywords:** Fluoride, oral microecology, ICDAS II, 16S rRNA sequencing, enamel initial caries

## Abstract

**Background:**

The caries-preventive effect of topical fluoride application has been corroborated by a number of clinical studies. However, the effect of fluoride on oral microecology remains unclear.

**Objective:**

To monitor the effect of fluoride on dental plaque microecology and demineralization/remineralization balance of enamel initial caries.

**Methods:**

Three-year-old children were enrolled and treated with fluoride at baseline and 6 months. International Caries Detection and Assessment System II indices of 52 subjects were measured at baseline, 3, 6, and 12 months. Supragingival plaque samples of 12 subjects were collected at baseline, 3 and 14 days for 16S rRNA sequencing.

**Results:**

Changes in microbial community structure were observed at 3 days after fluoridation. Significant changes in the relative abundance of microorganisms were observed after fluoride application, especially *Capnocytophaga*, unidentified *Prevotellaceae* and *Rothia*. Functional prediction revealed that cell movement, carbohydrate and energy metabolism were affected significantly after fluoride application. Fluoride significantly inhibited enamel demineralization and promoted remineralization of early demineralized caries enamel at 3 months.

**Conclusion:**

Fluoride application significantly inhibited the progression of enamel initial caries and reversed the demineralization process, possibly by disturbing dental plaque microecology and modulating the physicochemical action of demineralization/remineralization. This deepened our understanding of caries-preventive effects and mechanisms of fluoride.

## Introduction

Early childhood caries (ECC) is a chronic infectious disease characterized by progressive destruction of dental hard tissue with a high prevalence rate and rapid progression [1]. ECC frequently causes pain, masticatory discomfort, abscesses, and other symptoms, which may eventually lead to growth retardation, malocclusion, and poor quality of life in children [2]. Dental plaque biofilms are the primary drivers of ECC [3]. Normally, the microbial community of the biofilm is in dynamic equilibrium, and the solution around the tooth is supersaturated with respect to the tooth mineral so that the tooth does not demineralize [4,5]. However, with an increased frequency of sugar intake, cariogenic bacteria produce acid by glycolysis so that the pH value drops below the critical value (5.4–5.5). Afterwards, the surrounding solution is unsaturated, and the enamel crystal dissolves; thus, the enamel surface commences demineralization [6]. In contrast, symbiotic microorganisms intimately linked with oral health can counteract acidity by metabolizing ammonia and producing alkaline compounds; together with the buffer capacity of salivary and dental plaque, these phenomena combine to buffer the pH of the enamel surface close to neutral [5]. Moreover, the fluoride ions generated in the dissolution process and external mineral ions promote the redeposition of mineral salts of hydroxyapatite and other minerals on the surface, that is, remineralization [7]. Over time, white spot lesions or even caries lesions occur when the demineralization effect is greater than the remineralization effect. Fluoride varnish (FV) is a relatively widely used solution-type fluoride formulation that has been corroborated to be effective in reducing the risk of caries development in numerous *in vivo* and *in vitro* studies [8]. Fluoride ions can form a hybrid structure of fluorapatite (FA) and fluorhydroxyapatite (FHA) through reactions with hydroxyapatite on the enamel surface, thus reducing enamel solubility and inhibiting demineralization and promoting remineralization [9].

There are more than 700 bacterial species colonizing the human oral cavity, and most of them are unculturable [10]. Nonetheless, previous works on the consequences of fluoride on the oral microbiome have mostly relied on bacterial culture techniques and focused on only a few types of cariogenic bacteria [11]. For that reason, the effect of fluoride on oral microecology remains elusive. DNA-based methods, such as *16S rRNA* gene amplicon sequencing, have become a noteworthy strategy with wide applicability to investigate the composition and structure of the microbial community, especially in the detection of rare and yet-uncultured bacterial species [12]. It has been reported that topical application of casein phosphopeptide–amorphous calcium phosphate containing fluoride can modulate the microbial composition of the dental plaque microbiome towards symbiosis in children with caries by 16S rDNA-based next-generation-sequencing [13].

By comparison with the World Health Organization’s (WHO’s) diagnostic criteria, the International Caries Detection and Assessment System II (ICDASII) classifies and evaluates the status of dental caries based on visual examination and probing with sufficient specificity, sensitivity, and repeatability for the detection of enamel initial caries [14]. Several studies have previously investigated the effects of fluoride on oral microorganisms and the modulation of demineralization-remineralization balance in dental hard tissue. On the one hand, the effects on oral micro-ecology are likely transient and timeliness [15,16], which can be detected by the DNA-based methods in the short-term. On the other hand, the inhibitory effect on tooth demineralization and its promoting effect on remineralization are long-term and slow processes, which may take half a year and even longer to become detectable by the naked eye [17,18]. Therefore, this study aims to investigate the effect of FV on the microbial diversity of dental plaque biofilms of deciduous teeth by *16S rRNA* gene sequencing and to monitor the alterations of enamel initial caries lesions after fluoride application using the ICDAS II method. Based on the above process, we investigated the function and mechanisms of fluoride in caries prevention from the perspective of microbiology and enamel demineralization/remineralization, which led to further improvements in the use of fluoride for the prevention of dental caries in children.

## Materials and methods

### Subject selection

A random sampling method was designed and conducted to select 3-year-old children in a kindergarten as the study participants. The study inclusion criteria were as follows: (i) intact deciduous dentition; (ii) no pronounced tooth defects; (iii) freedom from congenital and systemic diseases; (iv) no topical application of fluoride (FV, fluorinated toothpaste, etc.); (v) voluntarily participated in the studies and follow-ups. The exclusion criteria were as follows: (i) developmental diseases of primary teeth; (ii) previous treatment of filling; (iii) antibiotic or immunosuppressant consumption within the past month; (iv) bacterial or viral infection in other parts of the body; and (v) inability to actively cooperate with sampling. Ethical approval was obtained from the Ethics Committee of the Stomatological Hospital, the Fourth Military Medical University (Xi′an, China). The ethical approval number is IRB-REV-2018065. The legal guardians of the subject children signed informed consent statements prior to the start of the study.

### Fluoride application

Fluoride application was implemented at specific time points (baseline and month 6). All dental surfaces were desiccated by sterile cotton swabs. Afterward, a small brush was dipped in FV (Duraphat®, Colgate, Germany) and used to coat dental surfaces. The subjects and their guardians were subsequently notified that they could not eat or drink water within 2 h and could not brush their teeth in the evening of the same day.

### Dental plaque sample collection

Supragingival plaque samples were collected at baseline (prior to fluoride application) and 3 days and 14 days after the first fluoridation. The children were instructed not to brush their teeth on the morning of the sample collection every time. Prior to sampling, they were requested to wash their mouths with sterile water, and saliva was subsequently wiped off with sterile cotton swabs. Pooled supragingival plaque samples were collected gently from deciduous teeth by means of sterile curettes and then deposited into sterile cryotubes containing PBS buffer and stored at −80°C. The triple samplings were successively labeled the H0 group, H3d group, and H2w group.

### DNA extraction, PCR amplification and 16S rRNA sequencing

Genomic DNA extraction and PCR amplification and purification were performed as previously described [19,20]. The primer-corresponding region was the 16S V4 region, with the forward primer 515 F (5’-GTGCCAGCMGCCGCGGTAA-3’) and reverse primer 806 R (5’- GGACTACHVGGGTWTCTAAT-3’). The library was constructed using an Ion Plus Fragment Library Kit 48 Reactions (Thermo Fisher, Germany). After the library was quantified by a Qubit Fluorometer (Thermo Scientific, Germany), the Ion S5^TM^XL platform was used for sequencing to obtain the V4 variable-region base sequence information of the 16S rRNA.

### Bioinformatics analysis

The raw sequencing data were preprocessed by Cutadapt software (version 1.9.1, http://cutadapt.readthedocs.io/en/stable/) [21] to acquire clean reads. The operational taxonomic units (OTUs) were clustered from clean reads using Uparse software (version 7.0.1, http://www.drive5.com/uparse/) [22] with a 97% similarity cutoff. Representative sequences of each OTU were singled out, and taxonomic information was annotated using the Mothur method (version 1.31.2) [23] against the SILVA132 database (http://www.arb-silva.de/) [24]. Alpha diversity indices were estimated using the QIIME software (version 1.9.1, http://qiime.org/scripts/split_libraries_fastq.html) [25] to evaluate the richness and diversity of microbial communities. Beta diversity indices based on weighted and unweighted UniFrac distance were calculated using the QIIME software, and principal coordinate analysis (PCoA) was performed using R software (version 2.15.3, Vienna, Austria) [26] to compare the dissimilarities in the microbial community composition and structure among groups at different time points. As a supervised multivariate statistical analysis method for discriminant analysis, partial least squares discriminant analysis (PLS-DA) performed by the R software could strengthen the difference between groups by adding grouping variables [27]. The Variable Importance in Projection (VIP) scores were combined to determine the key genera leading to the differential distribution of microbial community structure between groups (VIP > 1) [27]. Additionally, PICRUSt analysis was applied using the PICRUSt software (version 1.1.4, https://picrust.github.io/picrust/) [28] to predict genes and their metabolic functions in bacterial communities based on the KEGG database (http://www.genome.jp/kegg/) [29].

### Detection of ICDAS II scores

**Figure 1.**
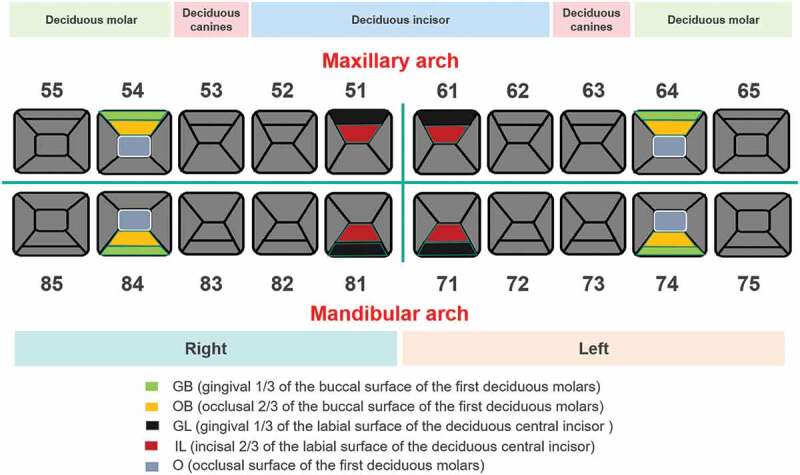
Detection teeth positions and sites using ICDASII. Representative positions and detected sites were selected, namely, the occlusal surface (O), gingival 1/3 of the buccal surface (GB), occlusal 2/3 of the buccal surface (OB) of the first deciduous molar teeth 54, 64, 74 and 84 and gingival 1/3 of the labial surface (GL), incisal 2/3 of the labial surface (IL) of the deciduous central incisor teeth 51, 61, 71 and 81.

The initial carious lesions were detected and diagnosed using the ICDAS II criteria at baseline and 3, 6 and 12 months after the first fluoride application. Among these time points, fluoride applications were implemented at baseline (subsequent to detection and sampling) and 6 months (subsequent to detection). Representative detected loci were selected, namely, the occlusal surface (O), gingival 1/3 of the buccal surface (GB), occlusal 2/3 of the buccal surface (OB) of the first deciduous molar teeth 54, 64, 74 and 84 and gingival 1/3 of the labial surface (GL), incisal 2/3 of the labial surface (IL) of the deciduous central incisor teeth 51, 61, 71 and 81 (Figure 1). The clinical examinations were performed by two trained pediatric dentists. The within-examiner correlation coefficient ranged from 0.59–0.88, and the kappa coefficient of the examiner themselves was 0.85.

### Statistical analyses

The statistical analyses of differences in sequencing data, alpha diversity indices, and predicted functional genes were performed by a paired Wilcoxon rank sum test. The statistical analysis of differences in beta diversity indices was assessed by Wilcoxon rank sum tests. The linear discriminant analysis (LDA) effect size (LEfSe) (v.1.1.2) analysis and LDA methods were implemented to identify the species with significantly differential relative abundances among groups at different time points. The LDA score threshold was set at 2. The Chi-square test was used to determine the statistical significance of differences in the proportion of ICDAS codes at different time points. The SPSS software (version 19.0, IBM, Chicago, IL) and LEfSe program (http://huttenhower.sph.harvard.edu/lefse/) [30] were used for conducting the statistical analyses, and P < 0.05 was considered statistically significant for all tests conducted.

## Results

### Study subjects

**Figure 2.**
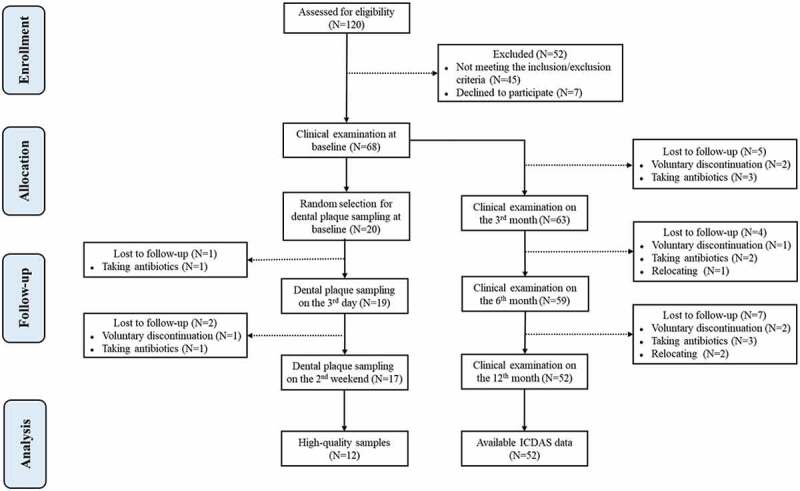
The CONSORT flow diagram of the progress through the phases of the trial (enrollment, allocation, follow-up, and analysis). N indicates the number of subjects. The number of lost subjects and the reasons for lost to follow-up are listed in the diagram.

A total of 120 subjects were screened, and 68 were subsequently enrolled in the study based on inclusion and exclusion criteria. Subjects were at a mean age of 3.4 ± 0.3 years old. The caries status of 68 subjects was assessed at baseline using the ICDASII. During the whole follow-up period, five subjects were lost to follow-up due to voluntary discontinuation, eight subjects were excluded due to taking antibiotics, and three subjects were excluded due to relocating (retention rate: 76%). Among them, 20 people were selected randomly to provide supragingival plaque samples at baseline. During the two-week follow-up period, two people were lost to follow-up due to taking antibiotics, and one subject was unwilling to participate. Ultimately, high-quality samples from 12 subjects were selected for 16S rRNA sequencing and bioinformatics analyses and the ICDAS II data from 52 subjects were used for statistical analysis. The CONSORT diagram is shown in Figure 2.

## Effects of fluoride on oral microecology

### Sequencing data and OTU analysis

A total of 2,439,060 clean reads with an average length of 412 base pairs were obtained after filtering from 36 samples, which accounted for 94.23% of the total raw reads (Table 1). The Good’s coverage was over 99.9% for all samples, illustrating that the current sequencing depth was sufficient to reflect the microbial diversity of dental plaques. The average numbers of taxon tags at baseline, 3 days and 14 days were 50,428 ± 11,464, 47,111 ± 12,003, and 51,659 ± 12,156, respectively, but no significant difference was observed by the Wilcoxon rank sum and Kruskal-Wallis tests (P > 0.05). The core microbiome could be found based on the common OTUs between the samples or groups and taxa that the OTUs represent. Among the 447,707 and 492 representative OTUs found at the three time points, 384 were shared among all groups, accounting for 49.2% of the total 781 OTUs sequenced from all dental plaque samples.

**Table 1.** Statistical table of reads, tags and OTU numbers of each sample from 16S rRNA sequencing. H0.1 ~ 12, H3d.1 ~ 12, and H2w.1 ~ 12 represent the samples at time points before, 3 days and 14 days after the first fluoride application, respectively.

Sample_name	Raw_reads(#)	Uniq_tag(#)	Tax_tag(#)	GC(%)	Q20	OTUs number(#)	Effective(%)
H0.1	56,915	12,214	41,452	52.78	82.73	252	94.29
H0.2	58,301	15,109	41,912	52.39	80.92	263	97.81
H0.3	63,507	12,608	45,059	53.88	81.94	192	90.80
H0.4	50,676	11,771	34,524	54.7	81.41	204	91.42
H0.5	53,079	10,277	41,684	56.16	83.18	197	97.89
H0.6	67,093	14,774	47,327	53.57	81.74	238	92.56
H0.7	64,214	12,159	46,702	53.72	82.41	236	91.67
H0.8	62,902	12,509	45,407	56.39	81.2	227	92.07
H0.9	90,713	19,597	63,866	52.53	82.19	305	92.01
H0.10	85,459	13,722	66,520	51.88	84.75	267	93.90
H0.11	83,755	15,589	64,550	51.38	85.06	290	95.68
H0.12	86,611	14,119	66,132	52.62	84.99	239	92.66
H3d.1	56,333	18,538	32,642	53.53	75.11	255	90.85
H3d.2	71,338	27,994	41,039	52.61	73.08	248	96.77
H3d.3	80,538	24,819	48,582	54.27	75.22	203	91.14
H3d.4	63,218	27,028	33,292	56.18	72.98	185	95.42
H3d.5	65,261	19,745	43,981	55.8	77.48	182	97.65
H3d.6	80,898	26,850	49,696	54.86	75.06	246	94.62
H3d.7	67,148	18,088	42,983	54.22	76.92	277	90.95
H3d.8	55,210	17,698	34,051	56.45	74.53	210	93.73
H3d.9	81,749	20,313	59,769	51.84	81.13	301	97.96
H3d.10	88,757	13,216	67,218	54.03	85.41	275	90.62
H3d.11	59,551	11,834	45,615	54.17	84.84	542	96.47
H3d.12	84,206	13,609	66,463	53.61	84.53	291	95.09
H2w.1	77,633	24,559	49,618	55.73	75.87	242	95.55
H2w.2	72,653	27,166	43,742	51.92	74.6	259	97.60
H2w.3	79,207	26,611	50,916	53.62	74.68	207	97.88
H2w.4	63,474	24,086	37,587	55.78	74.95	191	97.17
H2w.5	86,338	21,818	55,899	57.05	77.32	194	90.01
H2w.6	56,636	18,891	34,118	53.8	74.83	219	93.60
H2w.7	83,730	23,698	56,513	54.46	77	304	95.80
H2w.8	52,129	15,053	33,398	55.62	75.61	211	92.94
H2w.9	88,312	18,918	61,209	52.63	82.1	297	90.73
H2w.10	82,584	14,465	65,777	52.87	84.42	339	97.16
H2w.11	84,918	14,438	65,672	52.05	84.52	276	94.34
H2w.12	83,465	14,753	65,457	52.18	84.99	261	96.10

### Microbial diversity and community structure analysis

**Figure 3.**
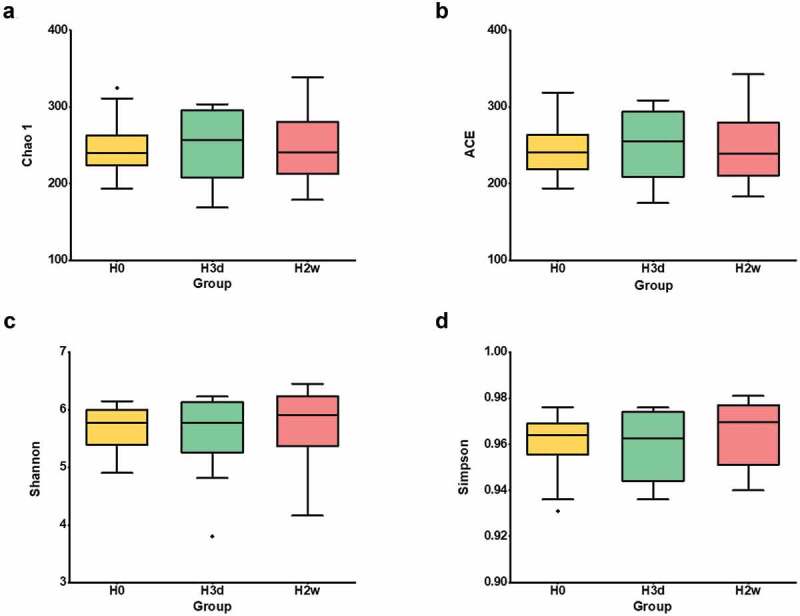
Alpha diversity analysis at different sampling time points. Alpha diversity indices of different samples at a 97% congruence threshold were statistically analyzed (**a**: Chao1; **b**: ACE; **c**: Shannon; **d**: Simpson), and the amount of data selected for homogenization was based on the cutoff = 32,438. H0, H3d and H2w represent the time points before, 3 days and 14 days after the first fluoride application, respectively.

**Figure 4.**
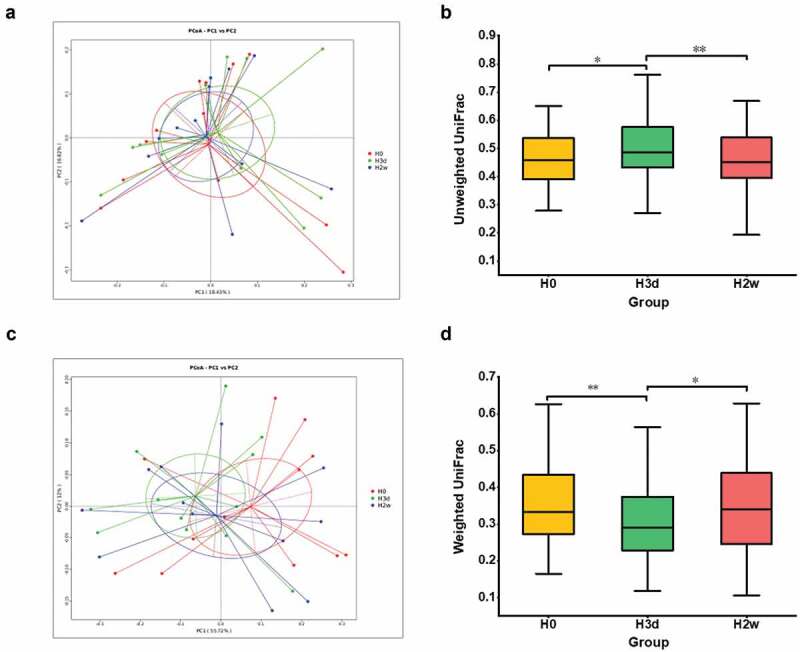
Beta diversity analysis at different sampling time points. Beta diversity were evaluated by PCoA analysis based on unweighted UniFrac distances (**a**) and weighted UniFrac distances (**c**). The data were analyzed by Wilcoxon rank sum tests (**b, d**). The asterisks indicate P values (one asterisk, P < 0.05; two asterisks, P < 0.01). H0, H3d and H2w represent the time points before, 3 days and 14 days after the first fluoride application, respectively.

Among the alpha diversity indices, the ACE and Chao1 index of richness showed a tendency of first increasing and then decreasing, and basically recovered to the baseline level on the second weekend. Meanwhile, the Shannon and Simpson of diversity indices displayed slight fluctuations. Despite these changes, no significant differences were observed among the three time points (P > 0.05) (Figure 3a-d). Beta diversity analysis with principal coordinate analysis (PCoA) based on unweighted UniFrac distance matrix indicated that little clustering with substantial overlap of the samples was observed at the three time points (Figure 4a), and Wilcoxon rank sum test showed that the unweighted UniFrac distance significantly increased initially (H0:H3d, P = 0.037), markedly decreased afterward (H3d:H2w, P = 0.0095), and progressively returned close to the original level (H0:H2w, P > 0.05) (Figure 4b), Additionally, PCoA analysis based on the weighted UniFrac distance matrix indicated that the samples of three time points clustered separately but with some overlap (Figure 4c), and Wilcoxon rank sum test showed that the weighted UniFrac distance significantly decreased initially (H0:H3d, P = 0.0062), markedly increased afterward (H3d:H2w, P = 0.0351), and progressively returned close to the original level (H0:H2w, P > 0.05) (Figure 4d). The above data indicated that fluoride had a significant disturbance effect on the microbial community structure of supragingival plaque, but with a minor effect on species richness and diversity.

### Taxonomic composition and differential abundance analysis

**Figure 5.**
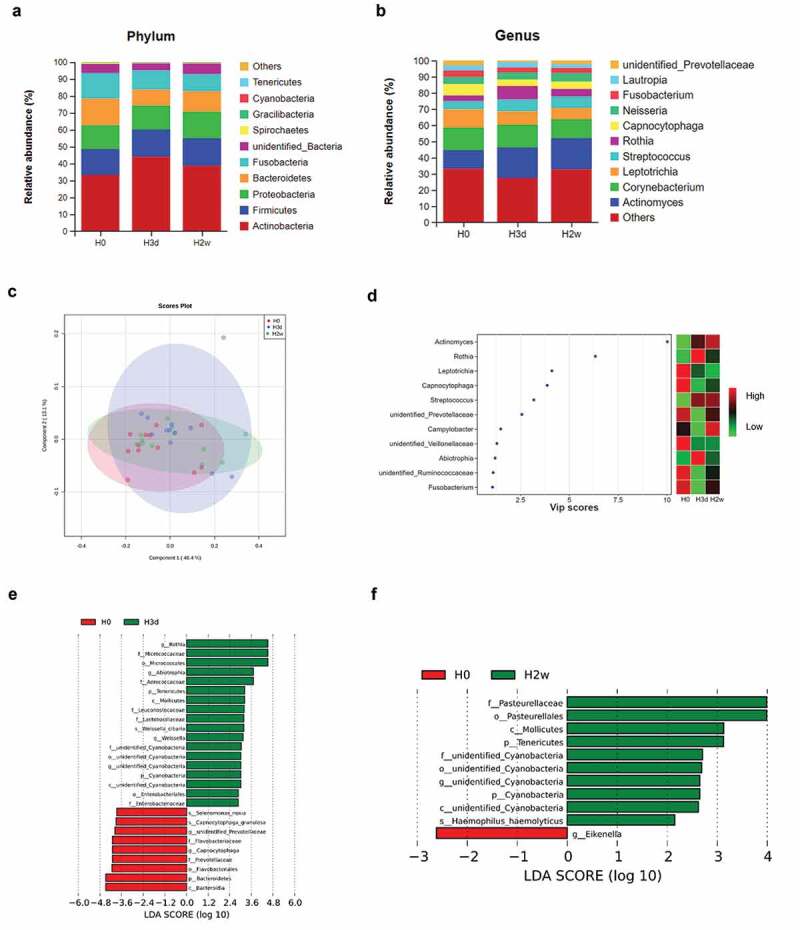
Bacterial abundance, distribution and differential microbiota compositions of supragingival plaque samples. Stacked bar charts showing the relative abundance of the supragingival plaque microbiota (the top 10) at the phylum level (**a**) and genus level (**b**) at three time points. The plot of PLS-DA analysis (**c**) indicates the difference between samples and groups, and the VIP scores plot of PLS-DA (**d**) reflects the key genera between groups at different time points. The abscissa represents the VIP scores and the ordinate represents the key genera with VIP scores >1. The histogram of LDA value distribution revealed the effect size of each differentially featured taxa (LDA > 2, P < 0.05) between baseline and 3 days (**e**) and between baseline and 14 days (**f**). Different colors suggest the enrichment of certain taxa in corresponding groups. H0, H3d and H2w represent the time points at baseline, 3 days and 14 days after the first fluoride application.

The OTUs were aligned with the SILVA132 database and could be annotated to 20 phyla, 29 classes, 58 orders, 111 families, 203 genera and 185 species. Among them, the phyla with relative abundance in the top ten in all groups were *Actinobacteria, Firmicutes, Proteobacteria, Bacteroidetes, Fusobacteria*, unidentified *Bacteria, Spirochaetes, Gracilibacteria, Cyanobacteria*, and *Tenericutes*, together accounting for more than 99.9% of the total sequences in each group (Figure 5a). The genera with relative abundance in the top ten in all groups were *Actinomyces, Corynebacterium, Leptotrichia, Streptococcus, Rothia, Capnocytophaga, Neisseria, Fusobacterium, Lautropia*, and unidentified *Prevotellaceae*, together accounting for 66.8%, 72.6% and 67.2% of the total sequences in each group (Figure 5b). The PLS-DA analysis showed that the samples grouped at three time points could be clustered separately with partially overlap (Figure 5c). A total of 11 bacterial genera were identified as the key genera causing significant differences in community composition (VIP > 1, Figure 5d). Among them, six genera played significant roles (VIP > 2), including *Actinomyces, Rothia, Leptotrichia, Capnocytophaga, Streptococcus*, and unidentified *Prevotellaceae*. The PLS-DA model combined with the VIP distribution map analysis could identify more bacteria with differential distribution among groups at different time points compared with LEfSe analysis. LEfSe analysis and LDA methods were performed to characterize the taxonomic alterations of the dental plaque microbiota in response to fluoride application. The results of LEfSe analysis with LDA methods for the different time points showed that the relative abundances of p_*Bacteroidetes*, c*_Bacteroidia*, o_*Flavobacteriales*, f*_Prevotellaceae*, g_*Capnocytophaga*, g*_*unidentified *Prevotellaceae*, and s*_Capnocytophaga granulosa*, s*_Selenomonas noxia* decreased significantly at day 3 (H0:H3d, P < 0.05, Figure 5e) and progressively returned to close to the original level at day 14 (H0:H2w, P > 0.05, Figure 5f). In comparison, a total of 18 species’ relative abundances were greatly increased at day 3, especially o_*Micrococcales*, f*_Micrococcaceae* and g*_Rothia* (H0:H3d, P < 0.05, Figure 5e). Among them, the relative abundances of p_*Tenericutes*, c_*Mollicutes*, p_*Cyanobacteria*, c*_*unidentified *Cyanobacteria*, o*_*unidentified *Cyanobacteria*, f*_*unidentified *Cyanobacteria*, g*_*unidentified *Cyanobacteria* continuously increased through day 3 to day 14 (H0:H3d, P < 0.05, Figure 5e; H0:H2w, P < 0.05, Figure 5f). Beyond these, 11 species’ relative abundances progressively returned to close to the original level at day 14 (H0:H2w, P > 0.05, Figure 5f). Compared to the baseline, the relative abundances of g_*Eikenella* was significantly reduced, and a total of 10 species’ relative abundances were significantly increased at day 14, especially f_*Pasteurellaceae*, o*_Pasteurellales* and c_*Mollicutes* (H0:H2w, P < 0.05, Figure 5f).

### Functional prediction analysis

**Figure 6.**
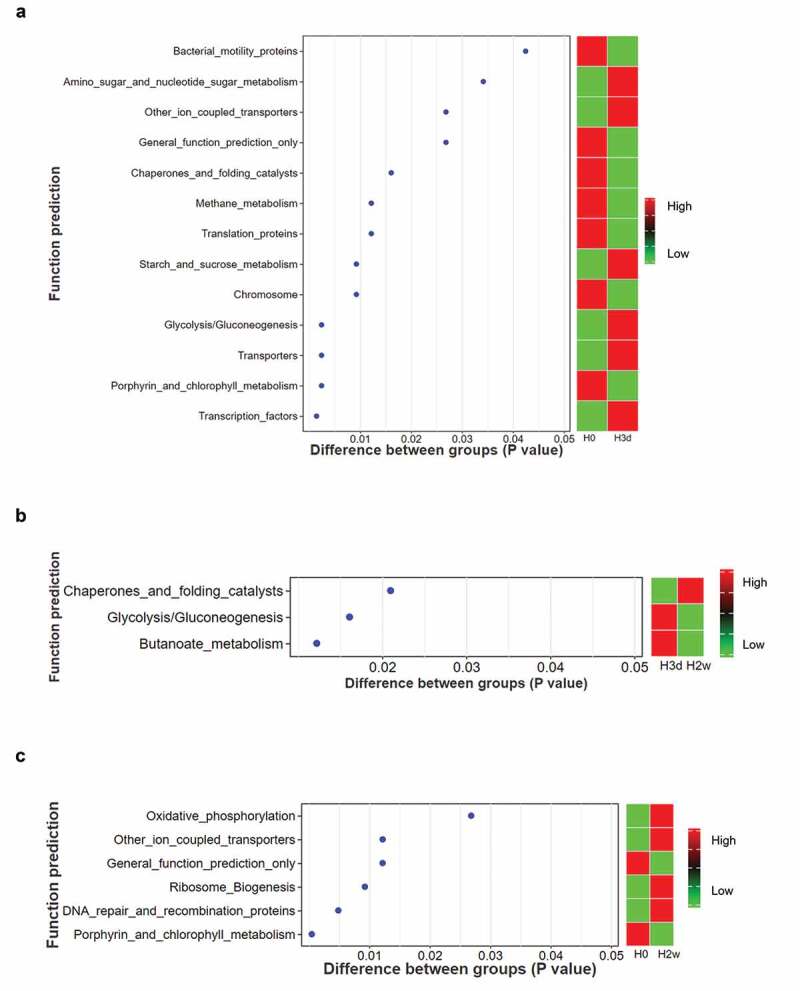
Analysis of significant differences in predicted metabolic functions between groups. Paired Wilcoxon rank sum test revealed the differences in predicted metabolic functions between baseline and 3 days (**a**), between 3 days and 14 days (**b**), and between baseline and 14 days (**c**). The abscissa represents the P values and the ordinate represents subfunctions of predicted metabolic pathways. P < 0.05 means statistically significant. H0, H3d and H2w represent the time points at baseline, 3 days and 14 days after the first fluoride application.

PICRUSt analysis showed that a total of 39 metabolic pathways at the secondary functional level were predicted, among which the most abundant metabolic pathways were membrane transport, carbohydrate metabolism, amino acid metabolism, replication and repair, translation, and energy metabolism. The paired Wilcoxon rank sum test revealed that the subfunctions of cell motility (bacterial motility proteins, P = 0.042), folding, sorting and degradation (chaperones and folding catalysts, P = 0.016), and genetic information processing (translation proteins, P = 0.012) were significantly depleted at 3 days post-fluoride application (Figure 6a). Nevertheless, the subfunctions of transcription (transcription factors, P = 0.001), carbohydrate metabolism (glycolysis/gluconeogenesis, P = 0.002; amino sugar and nucleotide sugar metabolism, P = 0.034; starch and sucrose metabolism, P = 0.009), membrane transport (transporters, P = 0.002) markedly increased from baseline to 3 days (Figure 6a). The subfunctions of carbohydrate metabolism (glycolysis/gluconeogenesis, P = 0.016; butanoate metabolism, P = 0.012) significantly decreased from 3 days to 14 days, and folding, sorting and degradation (chaperones and folding catalysts, P = 0.021) significantly increased over the same periods (Figure 6b). Furthermore, there was a trend toward significantly increased levels of the subfunctions of energy metabolism (oxidative phosphorylation, P = 0.027), replication and repair (DNA repair and recombination proteins, P = 0.005) at 14 days after fluoride application compared with baseline (Figure 6c).

### Effects of fluoride on early enamel caries detected by ICDAS II

**Figure 7.**
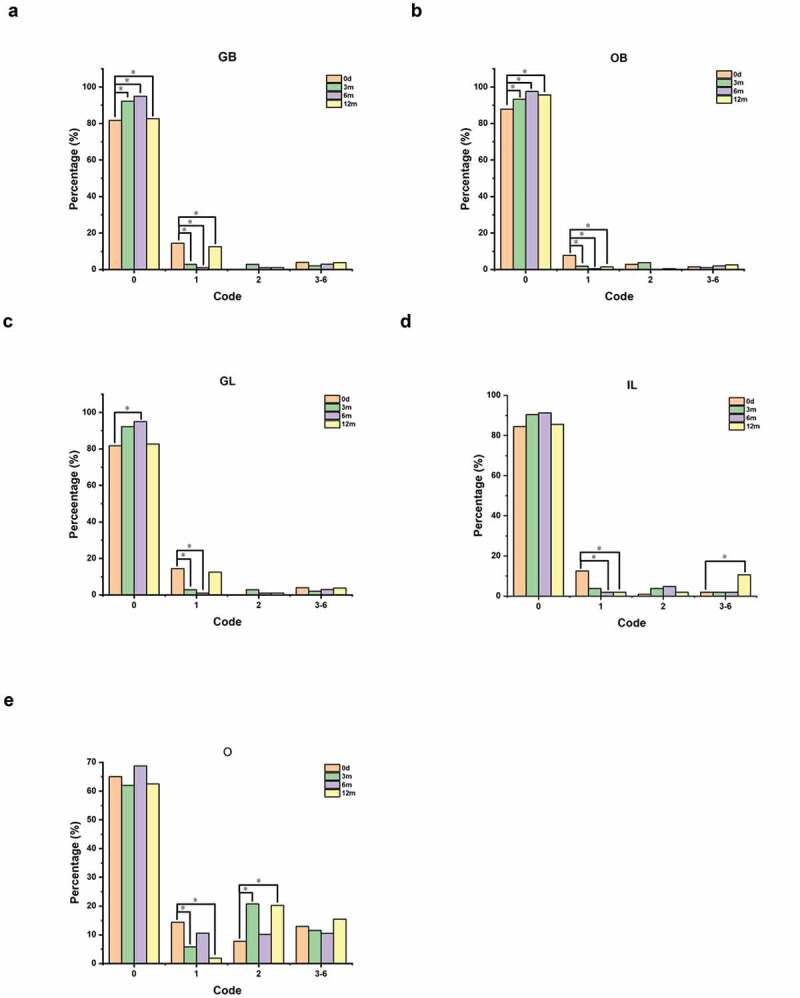
The proportions of different ICDAS II codes at different time points. **a-e** showed the change in the proportions of different ICDAS II codes at different time points at the sites of the gingival 1/3 of the buccal surface (GB, **a**), occlusal 2/3 of the buccal surface (OB, **b**), gingival 1/3 of the labial surface (GL, **c**), incisal 2/3 of the labial surface (IL, **d**) and occlusal surface (O, **e**). The abscissa represents the ICDAS II codes (0, 1, 2, 3–6) and the ordinate represents percentages of different codes at different time points. The asterisks indicate P values (one asterisk, P < 0.05),which represents the proportion of an ICDAS II code significantly different from the baseline.

Statistically significant differences were observed in the proportion of ICDAS II codes between baseline and each evaluation period (Figure 7). At sites such as gingival 1/3 of the buccal surface (GB) and occlusal 2/3 of the buccal surface (OB), there was a significant increase in the proportion of code 0 and a significant reduction in the proportion of code 1 (baseline vs. 3 months, baseline vs. 6 months, baseline vs. 12 months, P < 0.05, Figure 7a, b). At the gingival 1/3 of the labial surface (GL) site, there was a significant increase in the proportion of code 0 (baseline vs. 6 months, P < 0.05, Figure 7c) and a significant decrease in the proportion of code 1 (baseline vs. 3 months, baseline vs. 6 months, P < 0.05, Figure 7c). Nevertheless, at the site covering the incisal 2/3 of the labial surface (IL), the proportion of code 1 significantly decreased after fluoride application (baseline vs. 6 months, baseline vs. 12 months, P < 0.05), whereas the proportion of codes 3–6 significantly increased at 12 months (Figure 7d). At the site covering the occlusal surface (O), a significant drop in the proportion of code 1 and a significant elevation of code 2 were detected after each fluoride application (baseline vs. 3 months, baseline vs. 12 months, P < 0.05), even though they subsequently recovered (Figure 7e).

## Discussion

ECC is a multifactorial irreversible destructive oral disease with a high prevalence worldwide that is mediated by biofilms, driven by sugar, and evolving dynamically [31]. Untreated childhood caries usually leads to infection, pain, and poor oral health of children, which seriously endangers the children’s oral and general health [32]. Therefore, more attention should be devoted to the prevention and timely treatment of deciduous dental caries. Dental plaque is a microecological environment that colonizes the tooth surface in metabolization and pathogenicity, which is the initial and direct etiological factor of dental caries and periodontitis [33,34]. Currently, a large fraction of bacteria cannot be cultured and identified wholly by traditional techniques [35]. Molecular biology technology provides great convenience for microbiology research [36–39]. However, these techniques can only target dominant groups and cannot truly reflect microbial community composition and diversity. As one of culture-independent metagenomics detection technology, *16S rRNA* gene sequencing can reveal the composition and changes of oral microecology comprehensively through PCR amplification of the targeted region and strain identification [40]. In this study, the microbial diversity of supragingival plaque of deciduous teeth was detected by 16S rRNA sequencing, and 20 phyla, 29 classes, 58 orders, 111 families, 203 genera, 185 species and a large number of low-abundance or rare taxa were annotated, which was far more abundant than the microbial species detected by traditional technology. The data confirmed that 16S rRNA sequencing is an effective method for oral microbial research and can reflect the whole oral microecology in a relatively comprehensive way.

Microbial communities hold promise as a newly developing preventive and therapeutic breakthrough point for dental caries [41]. Under normal circumstances, owing to the interaction of symbiosis, competition and antagonism among microorganisms, the microbial community composition of the biofilm is in dynamic equilibrium [4,42]. However, due to the frequent intake of fermentable carbohydrates in the diet, the oral microbiota (especially cariogenic microorganisms) produces acid (mainly lactic acid) through the glycolysis pathway [5]. The acidification of the dental plaque biofilm microenvironment thus occurs, leading to the transition of microbial diversity and community structure and the occurrence and development of dental caries by prompting dental enamel demineralization [4]. Therefore, understanding how to regulate the changes in oral microecology provides insight into the prevention of dental caries [43]. Topical application of fluoride is currently one of the most extensively applied anti-caries measures. Considerable studies have shown that fluoride principally produces a marked effect in caries prevention by means of reducing enamel solubility, promoting enamel remineralization, and inhibiting bacterial metabolism [8,9]. Nonetheless, previous works on the consequences of fluoride on the oral microbiome have mostly relied on bacterial culture techniques and centered on only a few types of cariogenic bacteria, yet it remains unclear whether the application of fluoride causes changes in oral microecology. The 16S rRNA sequencing method was adopted in this study, and we found that the topical application of fluoride could ameliorate the oral microecological balance by impacting the species diversity, microbial community structure, and possibly affecting cell motility, carbohydrate, and amino acid metabolism, etc. The species richness and diversity appear to be an increasing trend at 3 days after fluoride application, which suggests that dental plaque microecology was more diversified after fluoride application. Additionally, beta diversity analysis based on weighted UniFrac distances revealed a significantly decreased dissimilarity index between samples at 3 days, which indicated that the community structure became more concentrated after fluoride application. It has been demonstrated that increasing species diversity, species homogeneity, and sample similarity mean healthier, more stable, and more complex microbial communities [44–46]. Moreover, fluoride affects the metabolic activities of dental plaque microorganisms, thereby improving the microenvironment of dental plaque [47]. Therefore, we speculate that the application of fluoride may contribute to the development of health-associated oral microbial communities in a time-dependent manner.

A previous study has evaluated the short-term effect of fluoride on the oral microbiota, and found that the microbial diversity of dental plaque changed within one week, whilst no significant change was observed in the microbial community at two weeks compared with the baseline [48]. In comparison, the long-term use of fluoride mouthwash had little effect on the adolescent oral microbiome composition during fixed orthodontic appliance treatment [49]. These findings are consistent with our data, in which the microbial community recovered close to its original level 14 days after fluoride application. Therefore, we speculate that the effect of fluoride on the oral microbial community is highly transient but effective. Dental plaque biofilms are composed of intricate microbial communities [41]. There may be a highly interactive, complicated network among microorganisms in dental plaque biofilms, which would generate a series of interdependences that furnish stability and resilience to change by symbiosis, synergism or antagonism [50]. The application of fluoride can temporarily disturb the dental plaque microbial community. However, the interactivities among microorganisms contribute to maintain community stability and resist change.

The oral core microbiome is of vital importance to the formation of dental plaque biofilms and has the potential to be a target for regulating the disequilibrium microecology of dental caries [51]. We herein revealed that most of the OTUs were shared by all groups, and 103 genera were distributed in all groups. They were defined as the core microbiome, of which the dominant genera were comparably abundant among all groups (relative abundance > 1%), such as *Actinomyces, Corynebacterium, Leptotrichia, Streptococcus, Rothia, Capnocytophaga, Neisseria, Fusobacterium, Lautropia* and unidentified *Prevotellaceae*. LEfSe analysis showed that a significant decrease in the relative abundance of unidentified *Prevotellaceae* and *Capnocytophaga*, and a significant increase in that of *Rothia* were observed after 3 days post-fluoride application. Meanwhile, PLS-DA/VIP analysis showed that unidentified *Prevotellaceae, Capnocytophaga*, and *Rothia* were identified as key genera which were responsible for significant differences in the community composition structure. It has been reported that unidentified *Prevotellacea* and *Capnocytophaga* are correlated to the occurrence and development of dental caries [52–54] and *Rothia* is associated with the oral health [55,56]. Moreover, the relative abundance of *Cyanobacteria* showed a gradually increasing tendency after fluoride application. *Cyanobacteria* have been reported to have antimicrobial potential in the oral environment, which deserves further investigation [57]. The present research revealed that fluoride might not only interfere with the growth of caries-associated bacteria, but also enrich the healthier dental plaque microbiota, which subsequently exert beneficial effect on the prevention and treatment of dental caries.

The ICDAS II system classifies and evaluates the status of dental caries in detail based on visual examination and probing, with high sensitivity and repeatability for the detection of dental caries, especially for early enamel caries [14]. Additionally, it effectively monitors the continuous changing process of dental caries [14] . To further evaluate the effect of fluoride on dental caries prevention from a clinical perspective, ICDAS II was used in this study to detect the changes in the degree of tooth surface mineralization after fluoride application. The results showed that a significant increasing trend of the constituent ratio of code 0 and a decreasing trend of code 1 were evaluated after fluoride application at almost all surface sites, which demonstrated that fluoride could significantly inhibit enamel demineralization and promote the remineralization of early demineralized caries enamel over the short term. Moreover, fluoride had a better caries-prevention effect on smooth surfaces than incisal edges of anterior teeth and occlusal surfaces of posterior teeth. These results were consistent with those of McDonald SP et al.; that is, the proportion of caries reduction on smooth surfaces was higher than that on occlusal surfaces after topical fluoridation [58]. We speculate that fluoride may exert its effect by driving supersaturation of the solution around the tooth and substantially accelerating the sedimentation rates of fluorapatite above the dissolution rates of hydroxyapatite. Additionally, remineralization occurs due to the deposition of newly formed minerals in the demineralized area, which leads to enamel initial caries lesions reversing to a certain extent [59].Caries lesions on the adjacent surface of anterior teeth frequently involve the incisal edge, which results in the inhibitory effect of fluoride not being pronounced enough on anterior teeth caries. These findings prompt us to apply other caries prevention measures in addition to topical fluoridation, such as strengthening anterior teeth adjacent to facial cleaning and implementing posterior teeth pit and fissure sealing as early as possible, hence preventing further expansion of dental caries.

In consideration of the fact that there were some variations in the subjects’ eating habits, we controlled the bias as much as possible by way of questionnaires and other measures. We acknowledged that the major limitation of the study was the small sample size and lack of the supragingival plaque sample of long-term fluoride application. Hence, further studies of greater sample size will be required to reduce the error caused by the differences within groups and to investigate the long-term effect of fluoride application on the oral microbiome. Additionally, the study lacked the supragingival plaque sample of teeth without fluoride application as a control. Some of changes in the oral microbiota due to time cannot be completely excluded. However, it is difficult to obtained the sample of teeth without fluoride application from the same kindergarten, because according to the recommendation from the Chinese Stomatological Association, the teeth of all kindergarten-aged children should be coated with fluoride varnish. As for the result of functional prediction analysis, PICRUSt is a predictive tool to predict potential metabolic pathways. The limitation of this approach should be considered in interpreting PICRUSt predictions. More work should be done based on functional analyses of metagenomics. Additionally, multiomics approaches that unify genomics, transcriptomics, proteomics, and metabolomics are indispensable to better understand the role of microorganisms in the initiation and progression of dental caries, thereby supporting theoretical underpinnings for effective biological control of dental caries [60].

## Conclusion

This study demonstrated that fluoride gave rise to a modest disturbance in the dental plaque biofilm microecology. Additionally, the regular application of FV twice per year conspicuously prevented caries and even reversed the development of initial enamel caries on smooth surfaces of deciduous teeth. The findings of the effect of fluoride on oral microecology provides insight into the mechanism of fluoride on caries prevention and opens up novel, effective and rational therapeutic avenues for the prevention and treatment of dental caries.

## Supplementary Material

Supplemental Material

## Data Availability

The datasets presented in this study can be found in online repositories. The names of the repository/repositories and accession number(s) can be found below: NCBI SRA BioProject, accession numbers: PRJNA831663 (https://www.ncbi.nlm.nih.gov).
