# Supplementary figures and images for: Application of fluoride disturbs plaque microecology and promotes remineralization of enamel initial caries
Source: J Oral Microbiol. 2022 Jul 27;14(1):2105022. doi: 10.1080/20002297.2022.2105022 (PMC9341347; doi:10.1080/20002297.2022.2105022)

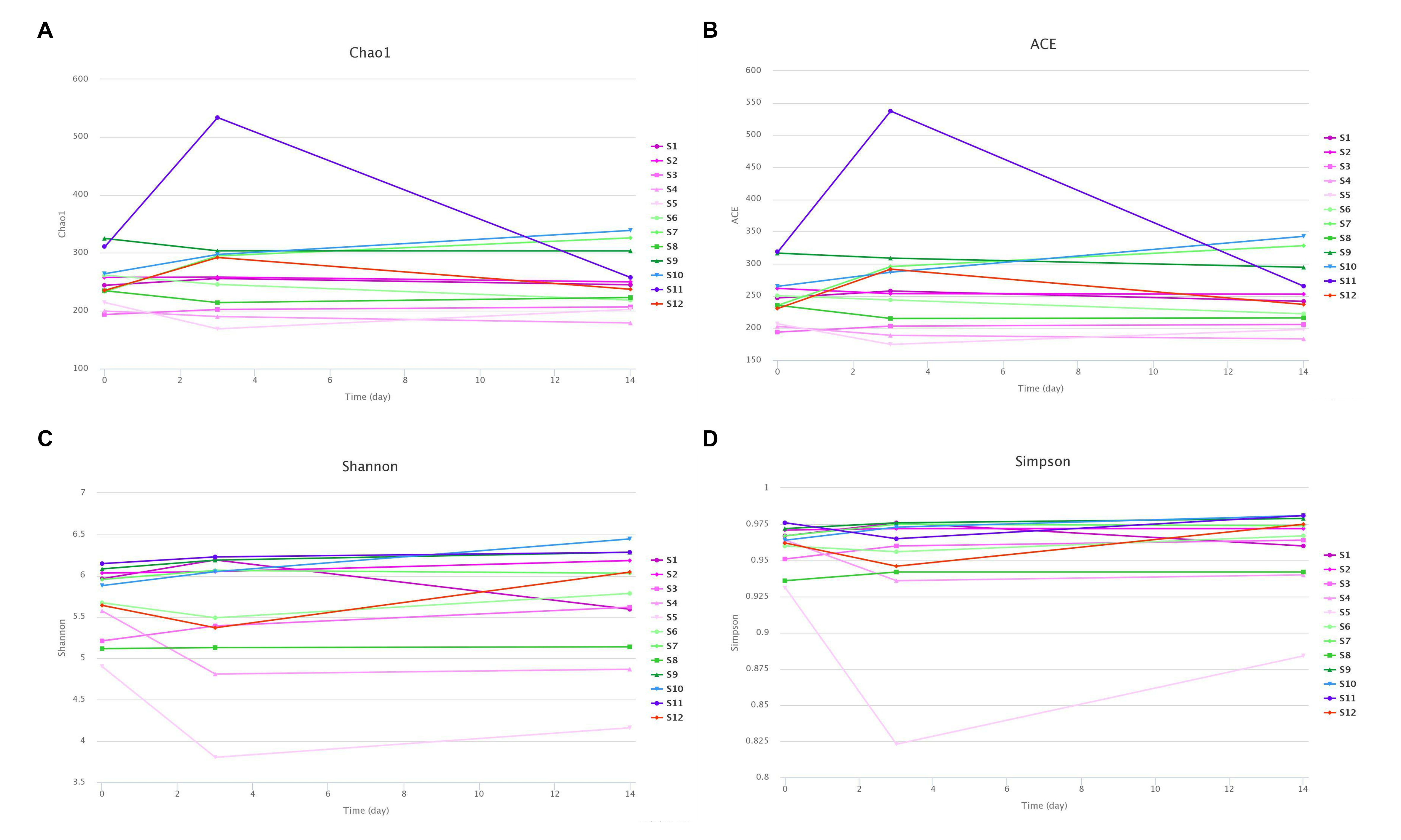

Supplement: Supplemental Material [file ZJOM_A_2105022_SM2111.zip › Supplementary files/Figure S1.tif]

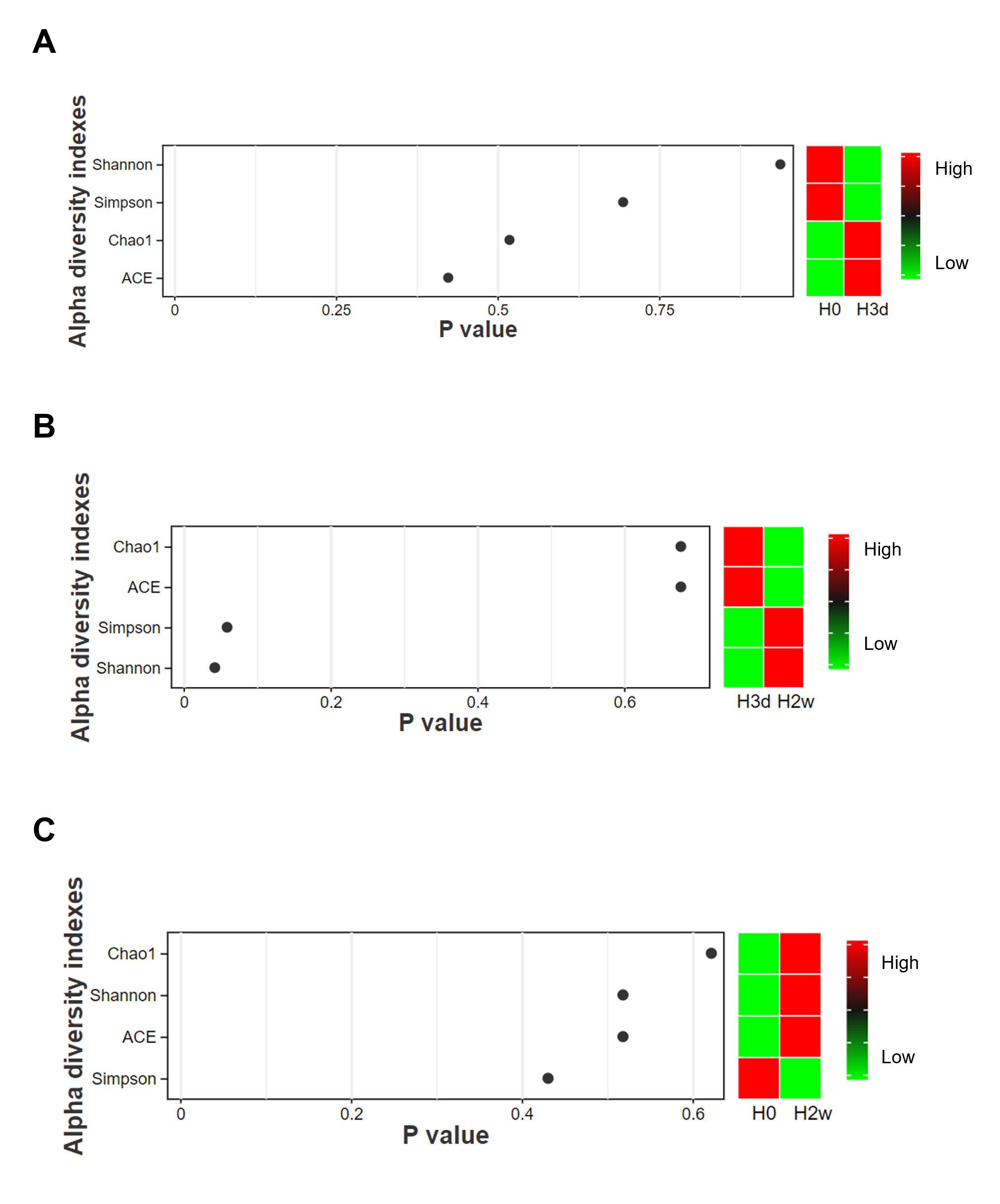

Supplement: Supplemental Material [file ZJOM_A_2105022_SM2111.zip › Supplementary files/Figure S2.tif]
